# Prognostic effect of programmed death-ligand 1 (PD-L1) in ovarian cancer: a systematic review, meta-analysis and bioinformatics study

**DOI:** 10.1186/s13048-019-0512-6

**Published:** 2019-04-30

**Authors:** Lin Wang

**Affiliations:** Women’s Hospital, School of Medicine, Zhejiang University, Hangzhou, 310006 China

**Keywords:** PD-L1, Ovarian cancer, Prognosis, Therapy

## Abstract

**Background:**

The expression of PD-L1 has been reported in ovarian cancer. However, the prognostic role of PD-L1 expression in ovarian carcinoma remained controversial. This study was performed to assess the prognostic value of PD-L1 expression on ovarian cancer.

**Methods:**

The PubMed, Embase, EBSCO, and Cochrane Library databases were searched to identify available publications. The pooled odds ratio (OR) or hazard ratios (HRs: multivariate analysis) with their 95% confidence intervals (95% CIs) were calculated in this analysis. A bioinformatics study based on The Cancer Genome Atlas (TCGA) sequencing and microarray datasets was used to further validate the results of PD-L1 mRNA expression. Kaplan-Meier (KM) survival curves were performed to evaluate the prognostic effect of PD-L1 mRNA expression.

**Results:**

Twelve studies with 1630 ovarian cancers regarding PD-L1 immunohistochemical expression were identified. Meta-analysis showed that PD-L1 protein expression was not associated with tumor grade, clinical stage, lymph node status, tumor histology, overall survival (OS), and progression-free survival (PFS). TCGA data showed no association between PD-L1 mRNA expression and ovarian cancer. Further validation using microarray data suggested that no association between PD-L1 mRNA expression and OS was found in large independent patient cohorts (1310 cases). PD-L1 mRNA expression was significantly linked to worse PFS in 1228 patients with ovarian cancer (227458_at: HR = 1.55, 95% CI = 1.28–1.88, *P* < 0.001; 223834_at: HR = 1.41, 95% CI = 1.14–1.75, *P* = 0.0015).

**Conclusions:**

Meta-analysis showed that PD-L1 may not be a prognostic factor for ovarian cancer. But a bioinformatics study showed that PD-L1 expression was significantly associated with worse PFS of ovarian cancer. More clinical studies are needed to further validate these findings.

**Electronic supplementary material:**

The online version of this article (10.1186/s13048-019-0512-6) contains supplementary material, which is available to authorized users.

## Introduction

Ovarian cancer is the second most common human gynecological malignancy and the most deadly gynecological malignancy among women [1]. According to global statistics, approximately 238,700 new cases were clinically diagnosed with ovarian carcinoma, and it killed 151,900 cases worldwide in 2008 [1]. Serous histology is the most common ovarian cancer, and other types consist of mucinous, clear cell and endometrioid carcinomas etc. [2]. Early stage patients with ovarian carcinoma generally have a favorable prognosis. However, most patients are diagnosed with advanced stages of this disease (stage 3–4), with a five-year survival rate of less than 20% [3, 4].

Programmed cell death receptor 1 (PD-1) belongs to the B7-CD28 family of costimulatory receptors and is expressed on the surface of T, B, and Natural killer (NK) cells that play key roles their activation and apoptosis [5, 6]. Programmed cell death-ligand 1 (PD-L1), also known as cluster of differentiation 274 (CD274) or B7-H1, is one of the PD-1 ligands and is expressed in on tumor cells and immune cells. PD-L1 is considered to be a crucial immunological escape mechanism that results in tumor cell growth, proliferation and metastasis [7–9]. Immune checkpoint blockade of PD-L1 with monoclonal antibody has shown promising approaches for improving survival rates of cancer patients [10]. PD-L1 expression has been reported to be associated with poor prognosis in many human cancers [11]. PD-L1 is also expressed in ovarian cancer. However, data regarding the prognostic effect of PD-L1 expression in ovarian cancer are limited, and some findings remain controversial. Hamanishi 2007 et al. reported that PD-L1 expression was correlated with poor overall survival in ovarian cancer [12]. But no association between PD-L1 expression and overall survival was found by Mills 2018 et al. [13].

To address the above-described issue, based on available publications, TCGA sequencing and microarray datasets, we conducted a systematic analysis to evaluate the relationship of PD-L1 expression with clinicopathological characteristics of ovarian cancer and the prognostic effect of PD-L1 expression.

## Materials and methods

### Systematic review

The PubMed, Embase, EBSCO, and Cochrane Library databases were systematically searched to identify all available studies using the following key words and search terms updated to September 16, 2018: ‘programmed cell death protein 1 OR PD-1 OR programmed cell death ligand 1 OR programmed cell death-ligand 1 OR CD274 OR B7-H1 OR PD-L1’, ‘ovarian OR ovary’, ‘cancer OR carcinoma OR tumor OR neoplasm’. In addition, a manual search from reference lists of all eligible studies was also conducted to get additional articles. This systematic review was conducted in accordance with the preferred reporting items for systematic reviews and meta-analyses (PRISMA) statement criteria [14].

The following inclusion criteria for the eligible publications were applied in this systematic review and meta-analysis: 1) the patients had a diagnosis of ovarian cancer based on histopathological examination; 2) cohort studies on the expression of PD-L1 using immunohistochemical (IHC) staining; 3) studies provided data to evaluate the relationship between PD-L1 expression and clinicopathological features of ovarian cancer patients (tumor histology, cancer grade, clinical stage, and lymph node status etc.); 4) studies reported sufficient information between PD-L1 expression and the prognosis of ovarian cancer using multivariate analysis, such as overall survival (OS) or progression-free survival (PFS). If authors published more than one paper using the overlapping population data, only the paper with more information was included in the current analysis.

The primary endpoint was OS, which was recorded as the time from the study enrollment to the date of death due to any cause or last follow-up. The secondary endpoint was PFS, which was defined as the time from the study enrollment until the first observed tumor progression or death.

The main exclusion criteria were as follows: 1) reviews, letters, case reports, or conference abstracts; 2) studies on cell lines and animals; 3) the detection method was not IHC; 4) studies lacking available data of PD-L1 expression and ovarian cancer, and 5) survival data using univariate analysis.

According to the selection criteria, author independently extracted necessary information from original articles in this systematic review and meta-analysis: first author’s surname, publication year, country, ethnic population, IHC staining, the frequency of PD-L1 expression, population size, effects on the clinical prognosis for multivariate analysis, and clinicopathological features such as tumor histology, tumor differentiation, clinical stage, and lymph node status etc..

### Bioinformatics study from TCGA sequencing and microarray datasets

The RNA-sequencing data and corresponding clinical information of patients with ovarian cancer were downloaded from The Cancer Genome Atlas (TCGA) (https://cancergenome.nih.gov/). Finally, 374 cancer patients with the available clinical data were identified. We determined the cut-off value of PD-L1 mRNA expression based on its median value. The Kaplan-Meier plotter tool (http://kmplot.com/analysis/) was also used to further analyze the clinical outcomes of PD-L1 mRNA expression using microarray data in ovarian cancer [29]. In this KM plotter database, PD-L1 mRNA expression data with OS information of 1310 ovarian cancer patients and PFS information of 1228 ovarian cancer patients were obtained.

### Statistical analysis

The overall odds ratios (ORs) with their 95% confidence intervals (95% CIs) were calculated for estimating the correlation between PD-L1 expression and the clinicopathological features of ovarian cancer. The combined hazard ratios (HRs) with their 95% CIs were used to determine the prognostic effect of PD-L1 expression in ovarian cancer. The heterogeneity among the eligible studies was detected using the Cochran’s Q test [15]. The random-effects model was used to make the results more reliable in the present study [16]. If substantial heterogeneity was detected for significant results (*P* < 0.1), a sensitivity analysis was performed to determine the influence of the pooled results by omitting an individual study [17]. For the results with greater than four studies, Egger’s test was used to measure the possible publication bias [18]. The pooled data from meta-analysis were analyzed using the Stata software, version 12.0 (Stata Corp., College Station, TX, USA).

The relationships between PD-L1 mRNA expression and the clinical characteristics were conducted by using the univariate logistic regression analysis. Survival curve was determined by Kaplan-Meier method and log-rank test. The univariate and multivariate Cox analyses were used to evaluate the role of PD-L1 expression on survival if possible. TCGA data were analyzed using R (v. 3.5.1, Institute for Statistics and Mathematics, Vienna, Austria). In the KM plotter database, OS and PFS of patients with ovarian cancer by a Kaplan–Meier survival plot with HR and log-rank *P* value were determined.

## Results

### Systematic review

**Fig. 1:**
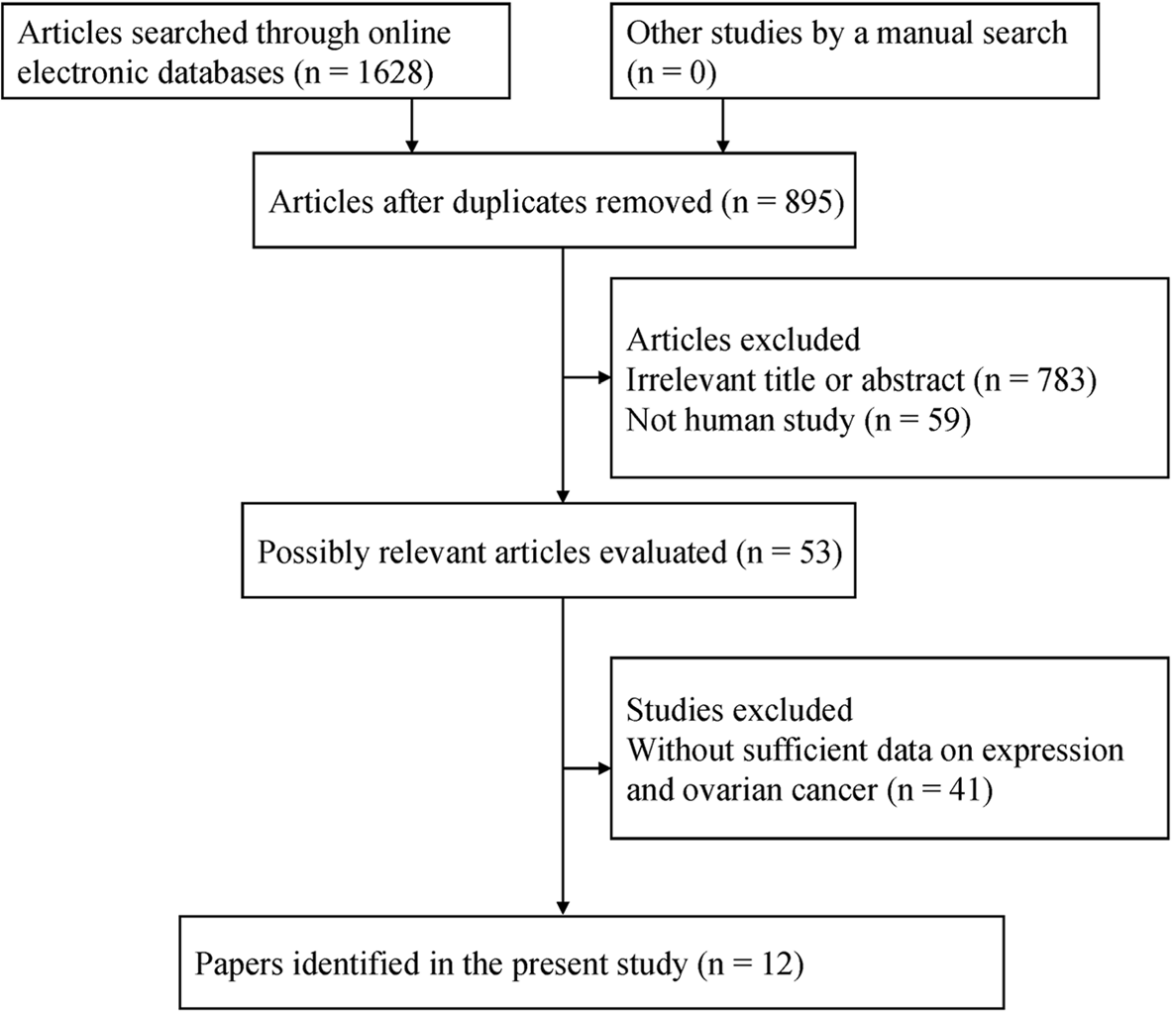
Flow chart of literature search and study selection

According to the above-described inclusion criteria and exclusion criteria, final 12 articles published from 2007 to 2018 [12, 13, 19–28] were identified in the systematic review and meta-analysis (Fig. 1), including 1630 patients with ovarian cancer. PD-L1 protein expression was detected using the IHC method. Nine studies evaluated the association of PD-L1 expression with clinicopathological characteristics of ovarian cancer [12, 13, 19, 20, 22–24, 26, 27]. Ten studies with 1525 ovarian cancer patients evaluated the prognostic role of PD-L1 expression using multivariate analysis [12, 13, 19, 21–25, 27, 28]. The main characteristics of the eligible studies are shown in Table 1.

**Table 1:** Main characteristics of the eligible studies from systematic review

First author	Country	Stage	Age	Abs	Cut off scores (IHC)	Cancer	Poor	Moderate/well	Stage 3–4	Stage 1–2	Node +	Node ^_^	Serous	Non-serous	Survival rate	Clinical outcomes
						N (E+ %)	E+/N	E+/N	E+/N	E+/N	E+/N	E+/N	E+/N	E+/N		
Hamanishi 2007 [12]	Japan	1–4	55	Clone 27A2	Scores 2 and 3	70 (68.6%)			29/39	19/31	11/14	37/56	21/28	27/42	5-year	OS, PFS
Darb-Esfahani 2016 [28]	Germany	1–4	60	Clone EPR1161	IRS 1–12	215 (93.9%)									5-year	OS, PFS
Webb 2016 [27]	Canada	1–3	NA	Cone SP142	1%	490 (34.9%)	108/278	63/212	37/83	134/407			112/206	59/284	10-year	DSS
Li 2017 [21]	China	1–4	NA	Ventana	Scores 3–6	140 (25%)									5-year	PFS
Mesnage 2017 [26]	France	2–4	NA	Clone E1L3N	5%	50 (30%)			14/49	1/1						
Chatterjee 2017 [25]	UK	NA	NA	Dako	Median	48 (NA)									5-year	PFS
Li 2017 [21]	China	1–4	NA	SP263	Scores 2 and 3	209 (15.8%)			15/128	18/81			13/113	20/95	5-year	OS
Wang 2017 [23]	China	1–4	57	Clone E1L3N	5%	107 (24.3%)			24/81	2/26					5-year	OS
Zhu 2017 [22]	China	1–4	53	ab205921	10%	122 (44.3%)			27/47	27/75					5-year	OS, PFS
Mills 2018 [13]	USA	1–4	NA	Clone SP142	1%	112 (28.6%)			26/92	6/19					3-year	OS
Drakes 2018 [20]	USA	1–4	61	ab205921	Scores 2–4	55 (32.7%)	17/41	1/13	13/46	5/9						
Zhu 2018 [19]	China	1–4	53	Clone SP142	10%	112 (58.9%)	10/16	46/80	41/70	25/42	20/31	37/63	45/75	21/37	5-year	DFS, OS

*IHC* immunohistochemistry, *NA* not applicable, *Ab* antibody, *E+* positive expression, *N* the number of the study population, *IRS* immuno-reactivity score, *OS* overall survival, *DS* disease-specific survival, *DFS* disease-free survival, *PFS* progression-free survival

**Fig. 2:**
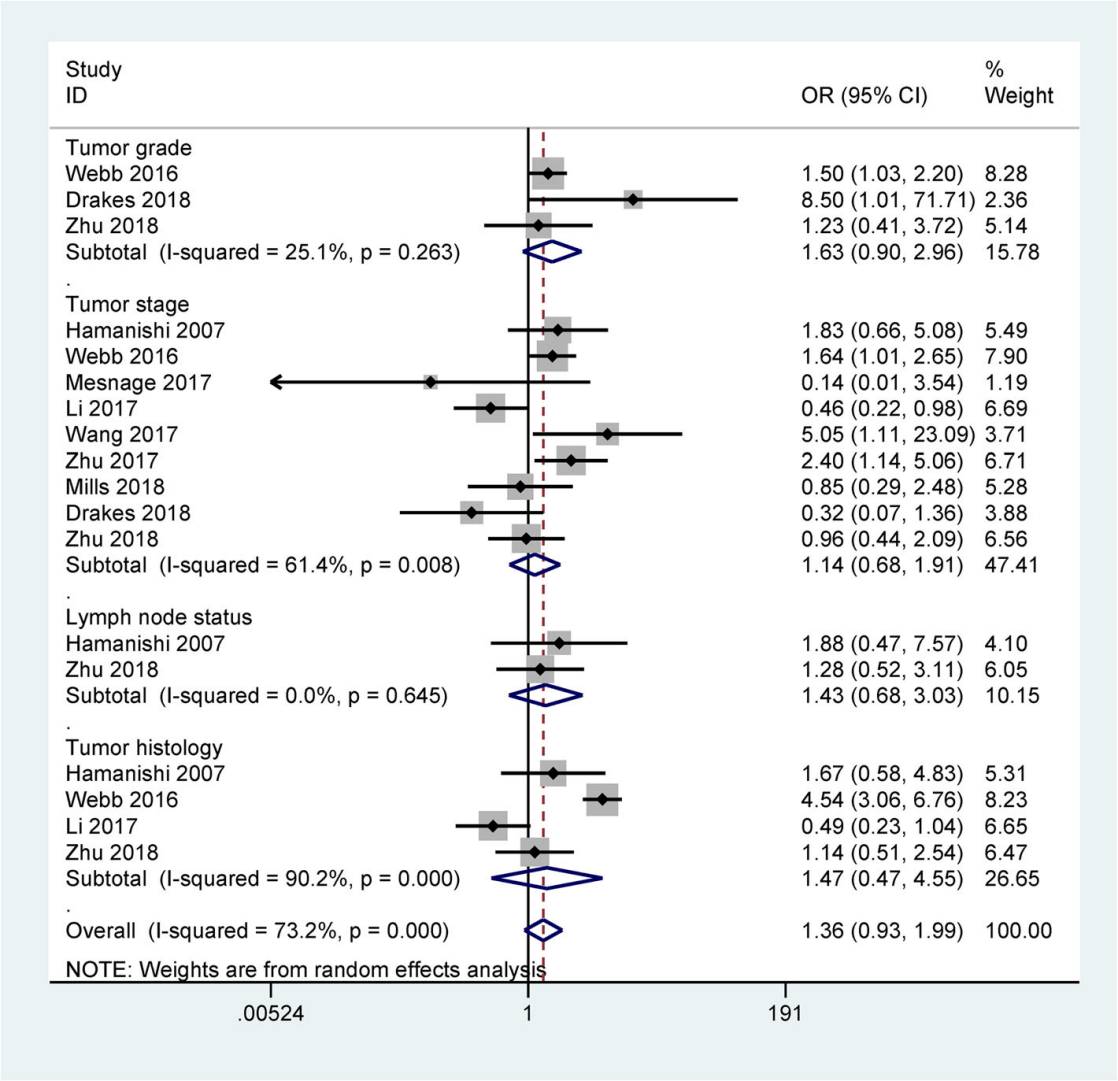
Forest plot for the relationship between PD-L1 protein expression and the clinicopathological features of ovarian cancer

The results of meta-analysis demonstrated that PD-L1 expression was not correlated with tumor grade (OR = 1.63, 95% CI = 0.90–2.96, *P* = 0.109, n = three studies with 640 ovarian cancer patients) and clinical stage (OR = 1.14, 95% CI = 0.68–1.91, *P* = 0.607, n = nine studies with 1326 patients with ovarian cancer) (Fig. 2). Data of meta-analysis from two studies with 164 ovarian cancer patients showed no association between PD-L1 expression and lymph node status (OR = 1.43, 95% CI = 0.68–3.03, *P* = 0.35) (Fig. 2). Meta-analysis showed that PD-L1 expression was not associated with tumor histology (OR = 1.47, 95% CI = 0.47–4.55, *P* = 0.507) (Fig. 2), including four studies with 880 ovarian cancer patients.

**Fig. 3:**
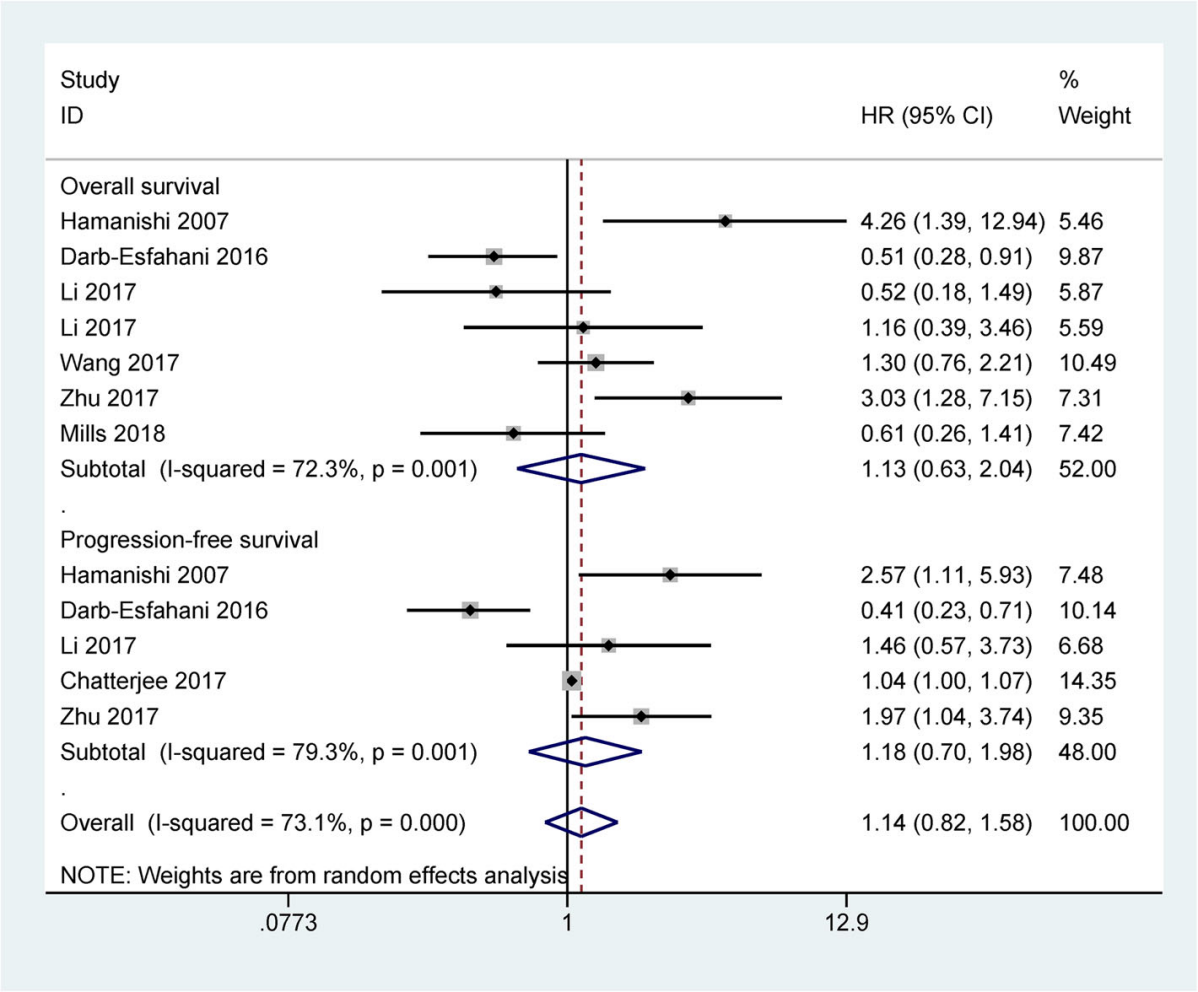
Forest plot for the association between PD-L1 protein expression and the prognosis

As shown in Table 2, only Zhu 2018 et al. reported that PD-L1 expression was not linked to OS and DFS in 112 ovarian cancer patients [19]. Only Webb 2016 et al. [27] reported that PD-L1 expression was correlated with favorable disease-specific survival (HR = 0.607, 95% CI = 0.399–0.925) in 195 patients with serous ovarian cancer. Based on qualitative analysis of DFS and disease-specific survival, more studies are needed to further validate the prognostic effect of PD-L1 expression in DFS and disease-specific survival in the future. Meta-analysis of long-term survival showed that PD-L1 expression was not linked to OS (seven studies with 835 patients: HR = 1.13, 95% CI = 0.63–2.04, *P* = 0.673) and PFS (five studies with 495 patients: HR = 1.18, 95% CI = 0.70–1.98, *P* = 0.532) (Fig. 3).

**Table 2:** Summary of clinical outcomes from systematic review

	Studies	HR with 95% CI	*P*_heterogeneity_	*P* values	N
Overall survival	7	1.13 (0.63–2.04)	0.001	0.673	835
Progression-free survival	5	1.18 (0.70–1.98)	0.001	0.532	495
Disease-specific survival	1	0.607 (0.399–0.925)	NA	0.02	195
Disease-free survival	1	NA	NA	> 0.05	112

*HR* hazard ratio, *95% CI* 95% confidence interval, *NA* not applicable, *N* the number of the study population

Heterogeneity of meta-analysis was found in relation to tumor stage (*P* = 0.008), when two studies [20, 24] were removed, heterogeneity was significantly reduced (*P* = 0.198) and the re-calculated OR was 1.56 (95% CI = 1.03–2.36, *P* = 0.037). Heterogeneity of meta-analysis was observed in relation to tumor histology (*P* < 0.001), when two studies [24, 27] were removed, heterogeneity was obviously decreased (*P* = 0.578). The pooled OR was not significantly changed (OR = 1.31, 95% CI = 0.69–2.48, *P* = 0.408).

Heterogeneity of meta-analysis was observed between PD-L1 expression and OS (*P* = 0.001). When we deleted three studies [12, 22, 28], the re-calculated HR was not significantly changed (HR = 0.92, 95% CI = 0.58–1.46, *P* = 0.727), with no heterogeneity (*P* = 0.287). Heterogeneity of meta-analysis was found between PD-L1 expression and PFS (*P* = 0.001). When we removed two studies [25, 28], heterogeneity was significantly decreased (*P* = 0.678) and the re-calculated HR was significant (HR = 1.98, 95% CI = 1.27–3.10, *P* = 0.003).

To examine the potential publication bias, Egger’s test was performed in the meta-analysis. The results showed that no publication bias was found between PD-L1 expression and tumor stage, OS, and PFS (all *P* values > 0.05) (Additional file 1: Figure. S1).

### TCGA and microarray datasets

The available clinical information were identified from TCGA for serous ovarian cancer. The univariate logistic regression analysis showed that PD-L1 mRNA expression was not significantly correlated with the clinicopathological characteristics of patients with ovarian cancer (Additional file 2: Table S1), including tumor residual disease (yes vs. no: OR = 0.75, 95% CI = 0.44–1.29, *P* = 0.297), cancer status (with tumor vs. tumor free: OR = 0.94, 95% CI = 0.57–1.54, *P* = 0.798), venous invasion (positive vs. negative: OR = 1.8, 95% CI = 0.81–4.02, *P* = 0.149), lymphatic invasion (positive vs. negative: OR = 1.67, 95% CI = 0.83–3.35, *P* = 0.147), tumor grade (poor vs. well or moderate: OR = 1.34, 95% CI = 0.7–2.53, *P* = 0.375), and clinical stage (stage 3–4 vs. 1–2: OR = 0.75, 95% CI = 0.32–1.76, *P* = 0.511). Kaplan-Meier survival showed that PD-L1 mRNA expression was not associated with OS in 374 patients with ovarian cancer (data not shown).

**Fig. 4:**
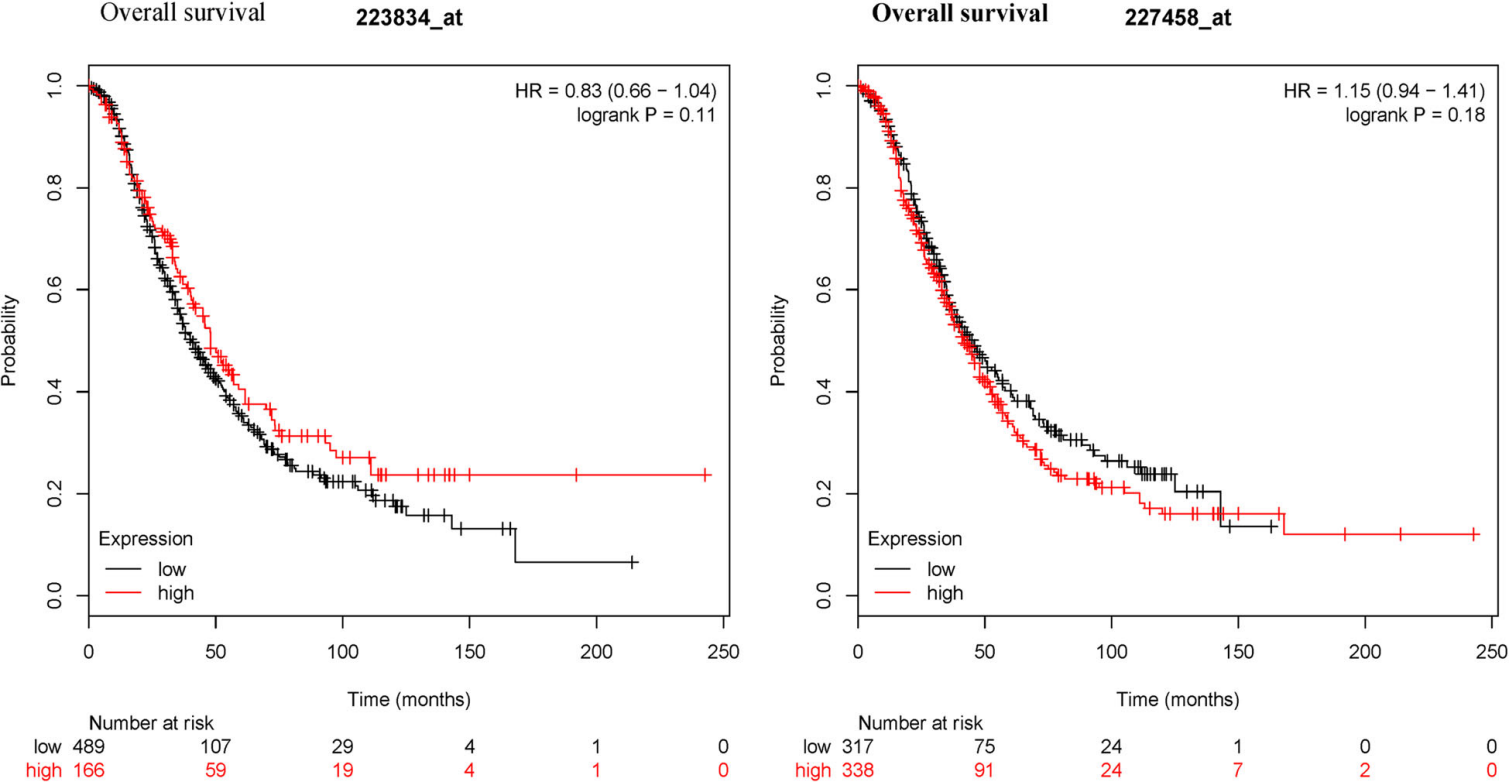
Kaplan-Meier plotter showing the prognostic role of PD-L1 mRNA expression in overall survival

**Fig. 5:**
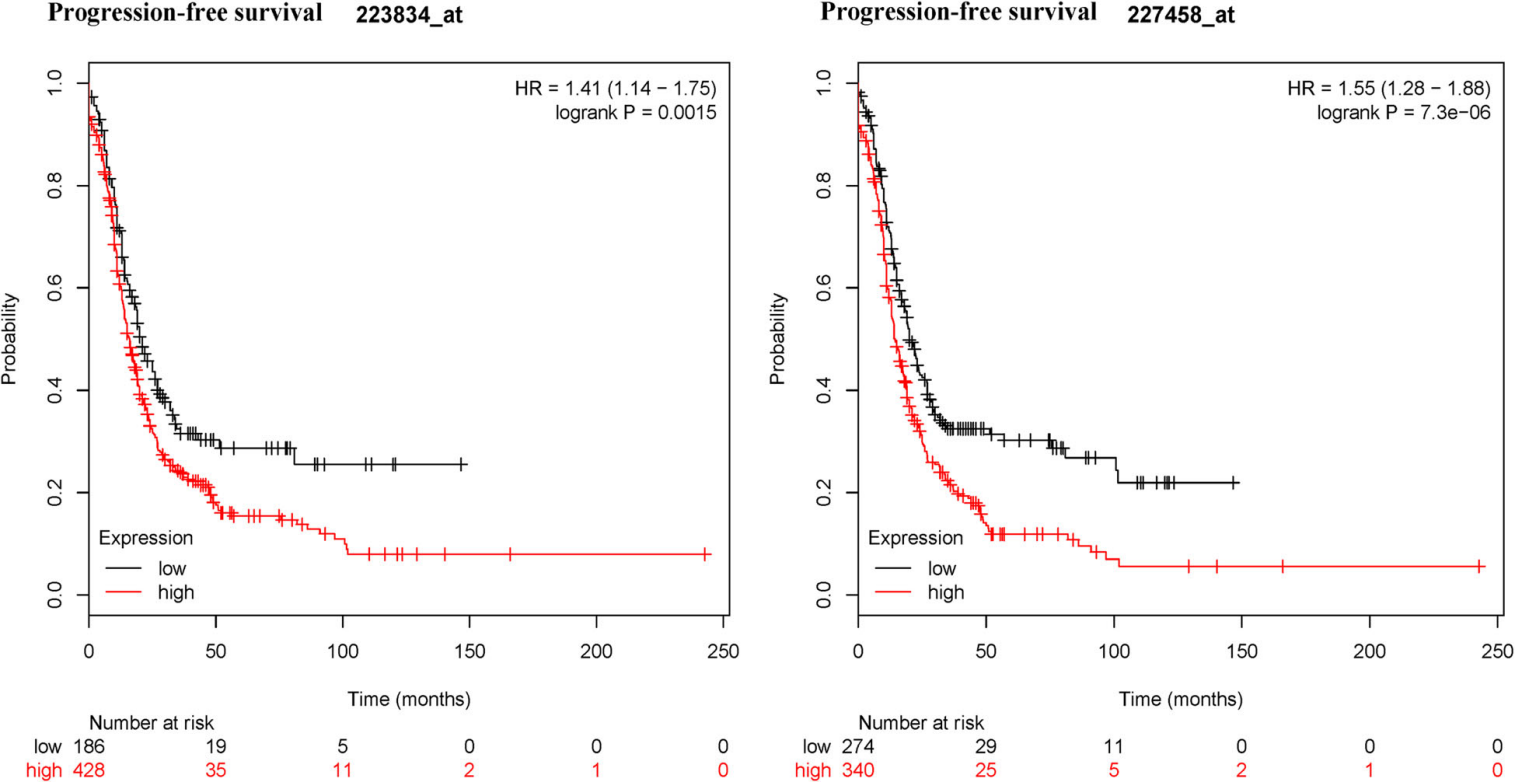
Kaplan-Meier plotter showing the prognostic role of PD-L1 mRNA expression in progression-free survival

An online KM plotter database using microarray dataset further showed that PD-L1 mRNA expression was not linked to OS in 1310 patients with ovarian cancer (227458_at: HR = 1.15, 95% CI = 0.94–1.41, *P* = 0.18; 223834_at: HR = 0.83, 95% CI = 0.66–1.04, *P* = 0.11) (Fig. 4). PD-L1 mRNA expression was significantly correlated with poor PFS in 1228 patients with ovarian cancer (227458_at: HR = 1.55, 95% CI = 1.28–1.88, *P* = 7.3 × 10^− 6^; 223834_at: HR = 1.41, 95% CI = 1.14–1.75, *P* = 0.0015) (Fig. 5).

## Discussion

Cancer immunotherapy is a novel approach of cancer treatment targeting the immune checkpoint receptors such as PD-L1. PD-L1 is an important immune regulatory factor and is closely correlated with the immune escape mechanism of cancer cells [30]. PD-L1 upregulation can be motivated by cytokines induced by tumor-infiltrating immune cells, including interferon (IFN), tumor necrosis factor (TNFalpha), interleukin (IL-4), and vascular endothelial growth factor (VEGF) etc. [31–34]. Blockade of the PD-L1 pathway is a promising immune-based treatment [35]. PD-L1 is expressed in many human cancers such as lung cancer [36], breast cancer [37], hepatocellular carcinoma [38], and cervical cancer [39]. PD-L1 expression in ovarian cancer has received great attention in recent years. At present, the association between PD-L1 expression and the prognostic role of ovarian cancer patients remains controversial [12, 13, 19, 21–25, 28]. Thus, the current study using available articles (this systematic review and meta-analysis), TCGA sequencing, and microarray datasets was conducted to evaluate whether PD-L1 expression was linked to different clinicopathological features and prognostic signature of patients with ovarian carcinoma. This work found that PD-L1 expression was significantly correlated with worse PFS, but was not associated with OS in ovarian cancer, suggesting that PD-L1 may serve as a useful prognostic marker for predicting PFS and serve as a therapeutic target in ovarian cancer.

The relationship between PD-L1 expression and the clinicopathological characteristics of ovarian cancer was analyzed. The results comprising all eligible studies with large populations showed that PD-L1 expression was not associated with tumor grade (poor differentiation vs. well/moderate differentiation, clinical stage (stage 3–4 vs. stage 1–2), and lymph node status (positive vs. negative), and tumor histology (serous vs. non-serous carcinoma) in this meta-analysis. Heterogeneity was observed between PD-L1 expression and clinical stage and tumor histology (*P* < 0.1). When we removed two studies [20, 24] in relation to clinical stage and two studies [24, 27] in relation to tumor histology. The results showed that heterogeneity was significantly reduced (*P* >  0.1), the re-calculated OR was not significantly changed for tumor histology, while the re-calculated OR indicated that PD-L1 expression was correlated with clinical stage (OR = 1.56, *P* = 0.037). The possible reasons of the heterogeneity remained unclear, perhaps because inappropriate conditions of immunohistochemical (IHC) staining may be used, which might lead to the observed bias in the present meta-analysis. Further bioinformatics data from TCGA showed that PD-L1 expression was not significantly associated with tumor residual disease, cancer status, venous invasion, lymphatic invasion, tumor grade, and clinical stage, which further suggested that PD-L1 expression was not correlated with advanced clinicopathological characteristics of patients with ovarian cancer.

Data from the present meta-analysis demonstrated that PD-L1 expression was not linked to the prognosis of ovarian cancer patients in OS and PFS. Heterogeneity was found between PD-L1 expression and the prognosis. When three studies [12, 22, 28] were removed in OS and two studies [25, 28] were removed in PFS. No heterogeneity was measured in OS and PFS. The re-calculated results indicated that PD-L1 expression was not linked to OS, while PD-L1 expression was significantly associated with worse PFS (HR = 1.98, *P* = 0.003). Further bioinformatics data from available microarray data validated that PD-L1 expression was not correlated with OS (*P* >  0.1) among a larger population (1310 ovarian cancer cases). While PD-L1 expression was significantly associated with unfavorable PFS (*P* < 0.001) in a larger population (1228 patients with ovarian cancer). Although OS is the most common gold standard endpoint. Recent studies have suggested that PFS has become an important and even a primary endpoint in clinical studies. In comparison to OS, PFS may be assessed using shorter and less costly studies [40, 41]. Therefore, the bioinformatics analysis further suggested that PD-L1 was a potential prognostic im-mune marker for ovarian cancer in PFS.

Several potential limitations should be addressed in this systematic review and meta-analysis. First, the search strategy was performed to obtain eligible studies published in English or Chinese. Other studies published in other languages, unpublished papers and conference abstracts were eliminated based on the difficulties in reading. Papers with positive results are more easily published than papers with negative results, which are lacking. These reasons may result in the potential bias and heterogeneity. Second, sample sizes regarding PD-L1 expression with the clinicopathological features of ovarian cancer were also small or absent such as lymph node status or distant metastasis, more studies should be further done in the future. Third, the results might have the potential bias due to the researcher’s viewpoint. To diminish this possibility, the study selection and data extraction were performed at least three times to ensure that all data are adequately truthful to the content of all cases included in this meta-analysis. Finally, additional prospective clinical studies should be done to further evaluate the prognostic effect of PD-L1 expression in ovarian cancer, especially for DFS and disease-specific survival.

## Conclusions

In summary, meta-analysis suggested that PD-L1 expression was not linked to tumor grade, clinical stage, lymph node status, tumor histology, OS, and PFS. A bioinformatics study demonstrated that PD-L1 mRNA expression was closely associated with poor PFS, which suggested that PD-L1 may become a promising therapeutic target for PFS of patients with ovarian cancer. To achieve more reliable conclusions, more prospective studies remain needed in the future.

## Additional files

Additional file 1: **Figure S1.** Publication bias using Egger’s test. (TIFF 454 kb)

Additional file 2: **Table S1.** Association of PD-L1 expression with the clinicopathological characteristics from TCGA dataset. (DOCX 14 kb)
