# Supplementary figures and images for: Prognostic effect of programmed death-ligand 1 (PD-L1) in ovarian cancer: a systematic review, meta-analysis and bioinformatics study
Source: J Ovarian Res. 2019 Apr 30;12:37. doi: 10.1186/s13048-019-0512-6 (PMC6492430; doi:10.1186/s13048-019-0512-6)

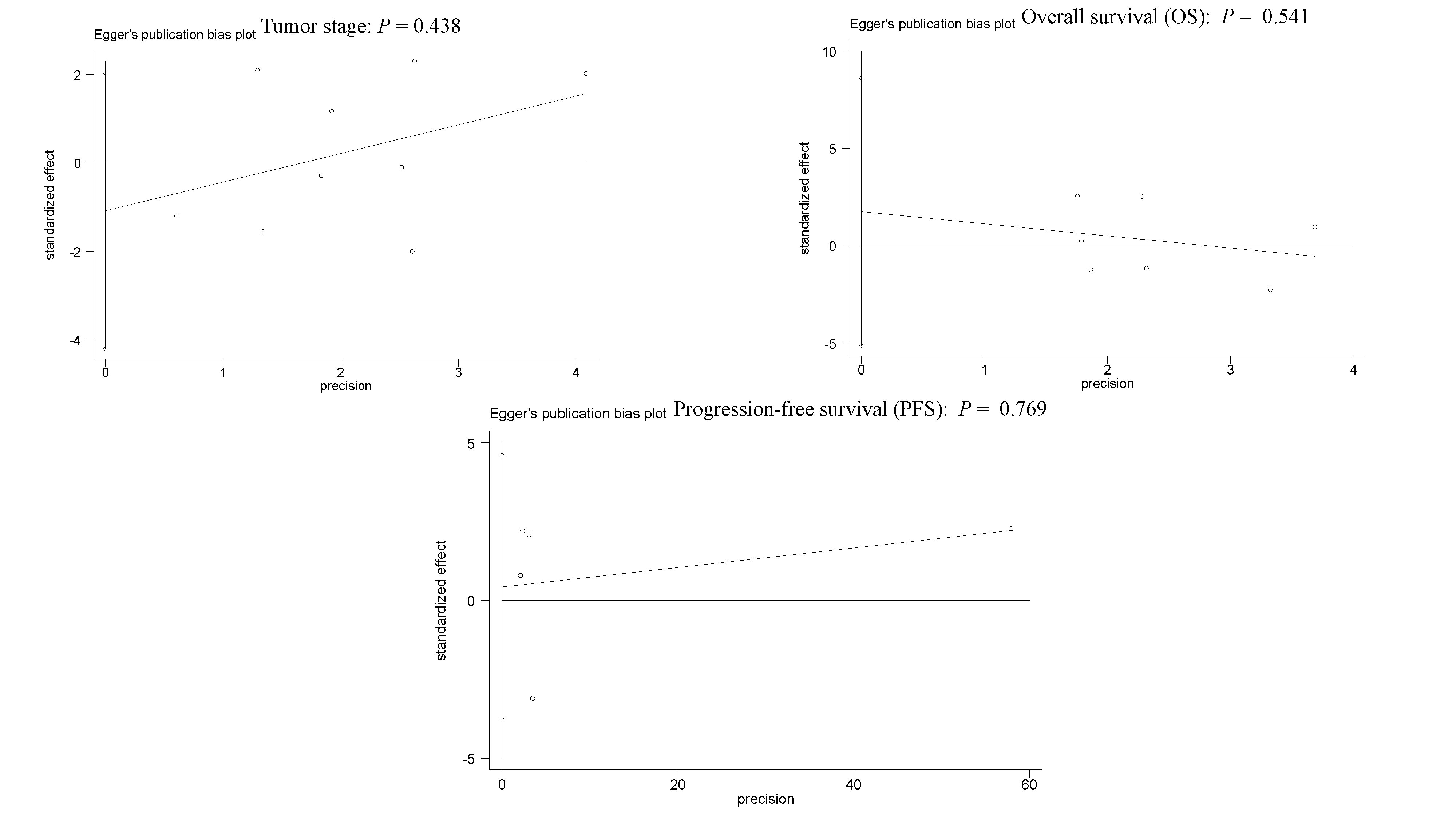

Supplement: Supplementary file 1 — Figure S1. Publication bias using Egger’s test. (TIFF 454 kb) [file 13048_2019_512_MOESM1_ESM.tiff]
