# Supplementary material for: Prognostic effect of programmed death-ligand 1 (PD-L1) in ovarian cancer: a systematic review, meta-analysis and bioinformatics study
Source: J Ovarian Res. 2019 Apr 30;12:37. doi: 10.1186/s13048-019-0512-6 (PMC6492430; doi:10.1186/s13048-019-0512-6)
Supplement: Supplementary file 2 — Table S1. Association of PD-L1 expression with the clinicopathological characteristics from TCGA dataset. (DOCX 14 kb) [file 13048_2019_512_MOESM2_ESM.docx]

Table S1. Association of PD-L1 expression with the clinicopathological characteristics from TCGA dataset

| Factors | Total (N) | OR with 95% CI | *P* |
| --- | --- | --- | --- |
| Tumor residual disease (Yes vs. no) | 332 | 0.75 (0.44-1.29) | 0.297 |
| Cancer status (With tumor vs. tumor free) | 330 | 0.94 (0.57-1.54) | 0.798 |
| Grade (Poor vs. well or moderate) | 364 | 1.34 (0.7-2.53) | 0.375 |
| Stage (3-4 vs. 1-2) | 371 | 0.75 (0.32-1.76) | 0.511 |
| Venous invasion (Positive vs. negative) | 103 | 1.8 (0.81-4.02) | 0.149 |
| Lymphatic invasion (Positive vs. negative) | 147 | 1.67 (0.83-3.35) | 0.147 |

N: number of the study population; OR: odds ratio; 95% CI: 95% confidence interval; TCGA: The Cancer Genome Atlas.
